# Risk Factors for the Comorbidity of Hypertension and Renal Cell Carcinoma in the Cardio-Oncologic Era and Treatment for Tumor-Induced Hypertension

**DOI:** 10.3389/fcvm.2022.810262

**Published:** 2022-02-17

**Authors:** Zhengqing Ba, Ying Xiao, Ming He, Dong Liu, Hao Wang, Hanyang Liang, Jiansong Yuan

**Affiliations:** ^1^Department of Cardiology, Fuwai Hospital, National Center for Cardiovascular Diseases, Chinese Academy of Medical Sciences and Peking Union Medical College, Beijing, China; ^2^Xiangya School of Medicine, Central South University, Changsha, China; ^3^Department of Infectious Diseases, Peking University First Hospital, Beijing, China; ^4^Key Laboratory of Pulmonary Vascular Medicine, Fuwai Hospital, National Center for Cardiovascular Diseases, Chinese Academy of Medical Sciences and Peking Union Medical College, Beijing, China

**Keywords:** hypertension, kidney cancer, comorbidity, targeted therapy, antihypertensive drug, cardio-oncology

## Abstract

Advances in tumor diagnosis and treatment, especially the use of targeted therapies, have remarkably improved the survival rate of patients with renal cell carcinoma (RCC), accompanied by higher hypertension (HTN) incidence among patients with RCC, reflecting the coming of a cardio-oncologic era. Therefore, for patients with RCC and HTN simultaneously, finding risk factors for the comorbidity and giving better clinical treatment have been urgent problems. In this review, we thoroughly investigated risk factors for the comorbidity of HTN and RCC based on preclinical and clinical studies. Firstly, RCC and HTN may have common risk factors, such as obesity, smoking, and other modifiable lifestyles. Secondly, RCC and HTN may lead to each other directly or indirectly by their therapies. We then discussed measures of reducing the comorbidity and treatment of HTN in patients with RCC. We also discussed the deficiency of current studies and pointed out future directions. In conclusion, this review aims to deepen the understanding of cardio-oncology and bring benefit to the population who are at high risk of getting or have already got RCC and HTN simultaneously.

## Introduction

The prevalence of hypertension (HTN) and renal cell carcinoma (RCC) keeps increasing. In 2019, one-third of people between 30 and 70 years old were estimated to have HTN globally and the number has doubled from 648 to 1.2 billion in the past 3 decades (1). HTN was the most frequent comorbidity with malignant tumors, seen in 38% of patients with cancer (2). RCC accounted for about 90% of renal malignancies (3). According to GLOBOCAN in 2020, the patients with kidney cancer were more than 1.2 million and new cases were estimated to be 431,288 globally (4). RCC prevalence in the United States was increasing owing to a higher incidence which had doubled compared with the incidence in 1975 (15.6 vs. 7.1 per 100,000 persons) and longer 5-year relative survival (75.6 vs. 52.3%), reported by the SEER program (5).

**Figure 1:**
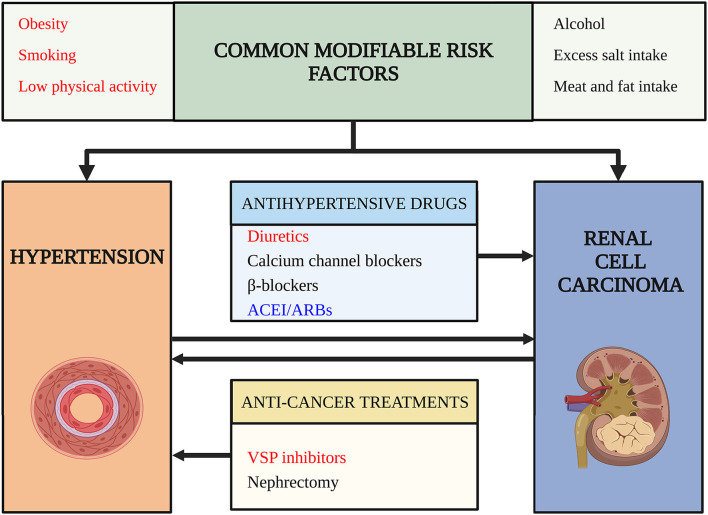
Risk factors for comorbidity of hypertension (HTN) and renal cell carcinoma (RCC). This figure outlines risk factors for the comorbidity of hypertension and renal cell carcinoma. Arrows indicate a potential causality relationship. Words in red color highlight risk factors confirmed by high-level evidence and have achieved consensus. Words in black color indicate risk factors lack strong evidence or the evidence are still conflictive. Words in blue indicate risk factors that may decrease the risk of comorbidity and have protective roles. ACEI/ARB, angiotensin-converting enzyme inhibitors/angiotensin receptor blockades; VSP, vascular endothelial growth factor signaling pathway.

Since the prevalence of HTN and RCC is increasing, patients with RCC and HTN simultaneously are estimated to increase for the following reasons: HTN is a potential risk factor for RCC (6) and RCC can cause HTN due to paraneoplastic syndrome (7), nephrectomy (8), and targeted therapies (9). Besides, prolonged survival rates and modern lifestyles may increase the comorbidity of HTN and RCC (10). The above-mentioned situation raised our questions: (1) What are the risk factors for the comorbidity of HTN and RCC in cardio-oncologic era? (2) How to decrease the comorbidity of HTN and RCC? (3) How to give better antihypertensive treatment for the patients with RCC with HTN? To answer these questions, we did a thorough search and reviewed the relationship between HTN and RCC based on clinical evidence and basic researches (Figure 1).

## Methods

A literature review of publications about RCC and HTN has been performed. A manuscript outline was formed before searching for relevant publications. PubMed (1946–2021) and Cochrane Library (1996–2021) were employed as the source of initial searches. Hand searching was also used to find relevant studies in PubMed and other websites (e.g., FDA and SEER). Besides, valuable publications recommended by experts were included as well. Key search words include HTN, antihypertensive agents, and kidney neoplasms. Detailed search queries and search results are available (Supplementary Material). In total, 7,279 studies were found. These studies were screened for eligibility using title and abstract. The remaining studies were then retrieved as full texts and checked with inclusion and exclusion criteria. We considered studies that were related to: (1) epidemiology about RCC or HTN; (2) risk factors causing RCC or HTN; (3) mechanisms for the formation of RCC or HTN; (4) treatment of HTN in patients with RCC. We excluded studies that were: (1) not in English; (2) duplicate; (3) clinical studies with similar results but lower evidence level or out of date; and (4) could not find full text. The review process is conducted independently by 3 authors. Discrepancies were solved by consensus.

## Common Modifiable Risk Factors

Obesity, inadequate physical activity and alcohol are well-known dose-dependent risk factors for HTN (11). The relationship between smoking and HTN is complex, but it is certain that cessation of smoking can dramatically reduce the cardiovascular disease burden (12). It is noteworthy that obesity, smoking, and inadequate physical activity are also risk factors for RCC (13) while alcohol exerts a protective effect on RCC development (14). Besides, diets play important roles in HTN and RCC. For example, excess salt intake increases blood pressure (BP) (11), whereas heavy meat and fatty food are risk factors for RCC, and lack of vegetables or fruits may also increase RCC incidence (15), but the common unhealthy diets for both RCC and HTN still need further study. In summary, obesity, smoking, and inadequate physical activity are common modifiable risk factors for RCC and HTN, we will discuss the clinical evidence and potential mechanisms below.

### Obesity as a Risk Factor for RCC

A meta-analysis of 24 cohort studies showed that the relative risk (RR) of kidney cancer was 35% higher (*RR* = 1.35, 95% CI = 1.27–1.43) in overweight [body mass index (BMI): 25–30] and 76% higher (1.76, 1.61–1.91) in patients of obesity (BMI > 30) compared with the normal weight population (16). Several indicators of obesity were used in clinical studies, such as BMI, waist and hip circumference, and body fat percentage, but the results were consistent (15). A large cohort study demonstrated that per unit increase in BMI will increase 5% risk of RCC (17). Both pre-existing obesity in adulthood and obesity near diagnosis of RCC could increase the risk of renal cancer [odds ratio (*OR*) = 1.6, same] (18). Of note, a cohort study in Japan demonstrated that low BMI (<21) may also increase the risk of kidney cancer [hazard ratio (*HR*) = 1.86; 95% CI: 1.01–3.45] compared with BMI of 23.0–24.9 (19). Interestingly, obesity was found to increase the risk of clear-cell RCC while decreasing the risk of papillary RCC (14). Such heterogeneity may be associated with demographic difference considering the fact that papillary RCC is more common in women, the older and the black (20).

Obesity-induced chronic renal hypoxia may play an oncogenic role mainly through upregulating the vascular endothelial growth factor (VEGF) pathway (21). Obesity could cause lipid peroxidation and then facilitate the formation of RCC (22). Obesity-induced renal hyperfiltration may increase the exposure to oncogenic nephrotoxins (23). Increased estrogen in adiposity patients also facilitates RCC by upregulating the insulin-like growth factor-1 (IGF-1) receptor, enhancing the oncogenic influence of IGF-1 (24). Metabolism disorders caused by obesity are also oncogenic. Overexpressed insulin and IGF-1 could promote the formation of RCC. Adiponectin, secreted by fatty tissue, is an anti-angiogenic factor by suppressing the VEGF pathway. However, the serum adiponectin is expressed lower in obesity (15). Besides, increased leptin in obesity, which is a kind of adipokine, promotes RCC by regulating VEGF, the Janus kinase/signal transducer and activator of transcription 3 and extracellular signal-regulated kinase 1/2 pathways (25). Obesity-induced inflammatory response increases levels of interleukin-6, which is also an oncogenic adipokine because it can protect RCC cells from immune attacks (26).

Insulin resistance and increased circulating insulin observed in obesity could induce HTN by increasing renal sodium reabsorption and activating the sympathetic nervous system (27). Elevated leptin can also promote HTN mediated by increasing sympathetic nervous system activity (28). Furthermore, a-melanocyte-stimulating hormone (a-MSH) that is a hormone secreted by melanocytes could regulate BP by suppressing adrenocorticotropic hormone. Interestingly, a-MSH could also inhibit obesity progress by targeting melanocortin 4 receptor. Therefore, it is hypothesized that sunshine may increase levels of a-MSH and then protect obese patients from inflammation-induced HTN (29).

### Smoking as a Risk Factor for RCC

A meta-analysis of 24 studies reported that smokers have a higher risk for RCC (*RR* = 1.38, 95% *CI*: 1.27–1.50) and a dose-dependent relationship was seen both in men and women (30). Remarkably, passive smoking increases the risk of RCC either (31). Cessation of smoking for more than 30 years reduced 50% risk of RCC (*CI*: 0.3–0.8), while quitting smoking shorter than 30 years showed no significant difference in RCC incidence (32). A retrospective study revealed that smoking is positively related to increased risk of clear-cell RCC rather than papillary RCC. However, this heterogeneity may be attributable to the skewed distribution of smoking patterns (14).

Some ingredients contained in cigarettes are carcinogenic. Nicotine could induce angiogenesis of RCC, while N-nitrosamines and Benzo[α]pyrene diol epoxide correlate with renal oxidative stress which could lead to DNA damage or gene aberration, thus facilitating the formation of RCC (15). Smoking-related chronic respiratory diseases and carbon monoxide could cause hypoxia of renal tissue (10) and lipid peroxidation is another possible mechanism (22).

Grassi et al. (33) found that smoking-induced acute increase of BP attributes to higher dose of catecholamines at the neuroeffector junctions. In addition, smoking was demonstrated to cause increased arterial wave reflection and stiffness of large arteries, thus enhancing BP (34). Smokers with atherosclerotic renal artery stenosis were more common than non-smokers, and renal vascular stenosis could cause refractory HTN (35).

### Low Physical Activity as a Risk Factor for RCC

Low physical activity is regarded as a risk factor for RCC (36–38). An American cohort study of 482,386 participants with a median follow-up of 8.2 years showed that the multivariate RR for those with current exercise more than 4 times per week was 0.77 (95% *CI*: 0.64–0.92) compared with the never exercise population. Besides, regular exercise and activity in youth is also protective (36). Similarly, a meta-analysis including 19 studies, which was conducted in 2013, showed that adequate physical activity was a protective factor for RCC (*RR* = 0.88, 95% *CI*: 0.79–0.97) (37). Physical activity like running or walking had a dose–response relationship with a decreased risk (1.9% risk decline per metabolic equivalents hour/week) of kidney cancer after adjustment for age and sex (38).

Physical activity may decrease the risk of RCC in a directly or indirectly manner. Physical activity could directly inhibit RCC formation by decreasing insulin resistance, circulating IGF-1, and lipid peroxidation (37). Some researchers thought the lack of exercise was an indirect risk factor because a low level of energy consumption could cause obesity and subsequently promotes RCC formation (13). Furthermore, more activity could prevent HTN and diabetes, which are also confounding factors (38).

The mechanisms for inactivity-induced HTN have not been clearly demonstrated. Murine studies showed that insulin resistance and imbalance of sympathetic and vagus nerves are potential reasons (39). Another animal study demonstrated that resistance training could contribute to the regulation of vessel constriction and keep luminal diameter (40). Other factors that explain inactivity-induced HTN include vascular resistance, arterial stiffness, oxidative stress, inflammation, BMI, and endothelial function (41).

## HTN as a Direct Risk Factor for RCC

A meta-analysis of 18 prospective studies and 14 case–control studies showed that each 10 mmHg increase of systolic BP (SBP) led to 5% higher risk (95% *CI*: 1.03–1.06) of RCC and 10 mmHg increase of diastolic BP (DBP) with 7% higher risk (95% *CI*: 1.04–1.10) (42). However, extremely high pressure (SBP > 150 mmHg or DBP > 100 mmHg) will cause a rapid increase of RCC incidence rather than linear growth (6, 43). It is noteworthy that even high-normal BP (SBP: 130–140 mmHg, DBP: 80–90 mmHg) could increase the risk of RCC (44).

Women with HTN may be more susceptible to RCC. A recent meta-analysis showed that women with HTN have a 54% higher risk than men (*RR* = 63 vs. 29%), but the difference was substantially reduced (1.40, 1.12–1.74 for men and 1.54, 1.17–2.04 for women) after adjustment for age, cigarette, family history of RCC, obesity, alcohol, and physical activity (42).

Age may influence the incidence of RCC in the patients of HTN while this hypothesis is still controversial. A study suggested that HTN was not an independent risk factor for RCC in adolescence (45), while another study got the opposite conclusion that younger patients with HTN were more likely to develop RCC (44).

It is worth mentioning that HTN may have a synergistic effect with obesity on RCC formation. A prospective study showed that the risk of obesity-caused RCC will increase significantly when BP was very high (SBP > 160 mmHg or DBP > 100 mmHg) (6).

A cohort study conducted in Sweden with a mean follow-up of 16 years among 3,63,992 men using repeated measurements of BP showed that RCC incidence decreased with the reduction of BP and especially, in those with a reduction of more than 14 mmHg in DBP, the RR for RCC decreased 40% (46). Thus, HTN is a modifiable risk factor for RCC and effective control of BP is of great value.

However, some factors may influence the reliability of these researches. HTN shares several common risk factors with RCC (43), which highlights the necessity of sufficient adjustment for these confounding factors during the investigation of the causality between HTN and RCC. Besides, the way of defining and measuring HTN varies (47). Of note, if RCC were diagnosed in patients with HTN in the first several years after enrollment in a cohort, it is difficult to determine the occurrence sequence of RCC and HTN. But such bias could be avoided by excluding data of the first several years of follow-up (46). In conclusion, well-designed prospective studies are warranted to clarify their causality.

As to the mechanisms of HTN-induced RCC, HTN could result in chronic inflammation, making the kidney in a state of hypoxia and then upregulating the expression of hypoxia-inducible factors, causing overexpressed VEGF and platelet-derived growth factors which could facilitate the tumor genesis (2). Overexpressed angiotensin receptors and angiotensin-converting enzyme in the patients with HTN could upregulate the angiotensin II, and cause the overexpression of oncogenic VEGF (42, 48). In addition, HTN is related to dysfunction and remodeling of blood vessels, which could increase the number of reactive oxygen species and eventually promote the formation and progress of tumor (44). Similar to obesity, an increased level of lipid peroxidation in the patients of HTN is supposed to participate in RCC carcinogenesis (22).

## Antihypertensive Drugs as a Potential Risk Factor for RCC

In general, antihypertensive drugs are not risk factors for cancers (49). However, a recent cohort study in Korea showed that the use of antihypertensive drugs in patients of HTN was related to increased risk of RCC (*HR* = 1.74, 95% *CI*: 1.64–1.84) and those with two or more classes of antihypertensive drugs have an even higher risk (*HR* = 1.80, 95% *CI*: 1.69–1.91) without adjusting for HTN (44). Another cohort study supported this result after adjusting for HTN, sex, age, BMI, and smoking (6). There seemed to be a linear relationship between the RCC incidence and the duration of antihypertensive drugs, and the risk will increase 2% per year (95% *CI*: 1.01–1.02) (50). However, different kinds of antihypertensive drugs play different roles in RCC development. Diuretics have been convincingly shown to be tumorigenic for kidney, while angiotensin-converting enzyme inhibitor/angiotensin receptor blockades (ACEI/ARBs) are possible anti-cancer drugs. The role of calcium channel blockers (CCB) and β-blockers is still in dispute. The tumorigenic role of these antihypertensive drugs will be elaborated below.

### Diuretics

Many researchers argued that diuretics are risk factors for RCC (47, 48, 50–53). A systematic review of observational studies in 2020 found that diuretics could increase 34% RCC risk (95% *CI*: 1.19–1.51) (50). Another meta-analysis showed that the risk effect of diuretics was still significant after adjustment for smoking, obesity, and HTN (47). Several cohort studies and case–control studies drew similar conclusions (48, 51). Women with diuretics (*OR* = 1.92, 95% *CI*: 1.59–2.33) seemed to have a higher risk of RCC than men (*OR* = 1.18, 95% *CI*: 0.93–1.49) (47). The sexual difference may be explained by the hypothesis that estrogens could intensify the effect of thiazide in the distal tubule, and women consume more diuretics than men (52). Some possible underlying mechanisms may explain the carcinogenic role of diuretics. First, hydrochlorothiazide could be converted in the stomach to nitroso derivatives and cause genetic mutations (53). Second, diuretics may exert a little carcinogenic function on their target, the renal tubular cells (51). More detailed preclinical studies are necessary to clarify the possible tumorigenic mechanisms of diuretics.

### Calcium Channel Blockers

The role of CCB on RCC carcinogenesis has not been determined yet (54). In patients without HTN, the use of CCB increased the risk of papillary RCC rather than clear-cell RCC, demonstrated by a retrospective study (55). CCB may predispose the patients with HTN to RCC by impeding DNA fragmentation and cell apoptosis (44). However, other clinical studies showed insignificant results which denied its carcinogenic role (54).

### β-Blockers

The role of β-blockers for RCC incidence is less well-studied. A recent cohort study showed that β-blockers have higher HR for RCC than other antihypertensive drugs (44). However, another large cohort study showed that β-blockers may not increase the risk of total cancer incidence (56). Thus, the exact role of β-blockers as a possible cancer-promotor is far from clear.

### Angiotensin-Converting Enzyme Inhibitors/Angiotensin Receptor Blockades

The role of ACEI/ARBs is still in dispute (57). A meta-analysis showed that ACEI increased the risk of RCC (*RR* = 1.50, 95% *CI*: 1.01–2.23) (58). ACEI may increase the amount of bradykinin which may facilitate RCC formation (57). Interestingly, ACEI/ARBs are also considered as possible anti-cancer drugs since overexpressed angiotensin receptors and angiotensin II is associated with upregulated VEGF (59).

Even though many clinical studies have managed to clarify the causality between antihypertensive drugs and RCC, there are still no conclusive results because of some limitations. Firstly, it is quite difficult to exclude the effect of HTN *per se* (10). For example, a large prospective study in 2008 showed that in those with SBP < 160 mmHg or DBP < 100 mmHg, the use of antihypertensive drugs did not show a significant difference compared with non-users while in those with poorly controlled BP, antihypertensive medication increased the risk of RCC, which highlighted the confounding role of HTN (6). Secondly, other confounding factors like age, sex, obesity, smoking, and physical activity are sometimes not adjusted because of the small sample size or poor statistical design. Thus, a well-designed large prospective clinical study is needed to clarify the relationship between antihypertensive drugs and RCC.

## RCC Directly Cause HTN

The HTN directly caused by RCC is considered as a manifestation of paraneoplastic syndrome and in the population with malignant HTN, the prevalence of RCC was 1.2%, much higher than those without malignant HTN (0.01%) (7), indicating malignant HTN could be a clue for the diagnosis of RCC. The severity of paraneoplastic HTN varies and can sometimes cause refractory HTN. Most of the paraneoplastic HTN will recover after nephrectomy (60–62).

Tumor compression, renal arteriovenous fistula, and ureteral obstruction could cause renal ischemia, thus activating the rein-angiotensin-aldosterone system, leading to HTN (63, 64). Besides, ectopic hormones secretion, such as catecholamines, erythropoietin correlated with paraneoplastic HTN (7, 60). Hypercalcemia, which increased vascular resistance or indirectly increased catecholamines, could also cause HTN (61, 62). In addition, paraneoplastic nodular polyarteritis correlated with renal vascular HTN (59). It is rarely reported that brain metastasis from RCC could cause intracranial HTN by compressing dural venous sinuses (65).

## Treatment of RCC Cause HTN

The treatment of RCC mainly includes surgery for localized RCC, targeted therapy, and immunotherapy for metastatic RCC (mRCC) (3). The excision of kidney jeopardizes kidney function and then increases the risk of cardiovascular disorders, such as coronary heart disease, HTN, cardiomyopathy, heart failure (HF), and dysrhythmias (66). Considering that partial nephrectomy (PN) can better preserve kidney function than radical nephrectomy (RN), PN is recommended to treat patients with early stage tumors (3). Nephrectomy-related HTN (NR-HT) has been reported by several studies, but robust high-level evidence is still needed (8, 67–69). The use of targeted therapies, especially vascular endothelial growth factor signaling pathway (VSP) inhibitors, has remarkably increase the life expectancy of patients with mRCC while the increased risk of cardiovascular events turns out to be its obvious side effect (5, 70). Apart from HTN, VSP inhibitors could also cause venous thromboembolism (VTE), HF, arterial thromboembolism (ATE), myocardial infarction (MI), long Q-T syndrome (LQTS) and Torsade de Pointes (TdP). Detailed information is listed in Table 1. Immnunotherapy is also a first-line therapy for mRCC but significant cardiovascular side effects have not been found yet (3, 70).

**Table 1:** Incidence of targeted therapy associated hypertension (HTN) in patients with metastatic renal cell carcinoma (mRCC).

**Drugs**	**FDA approved year**	**Any grade HTN (%)**	**Grade 3/4 HTN (%)**	**Other associated cardiovascular complications**
Temsirolimus	2007	7	-	VTE, thrombophlebitis
Everolimus	2009	1–10	3	Non-infectious pneumonitis with pulmonary HTN, VTE, tachycardia, HF
Bevacizumab	2004	4–34	1–11	ATE, VTE, HF
Sorafenib	2005	12–34	4–11	MI, LQTS
Sunitinib	2006	24–41	8–15	MI, HF, cardiomyopathy, LQTS, TdP
Pazopanib	2009	13–57	4	LQTS, TdP, HF, ATE, VTE, thrombotic microangiopathy
Axitinib	2012	40–42	8–16	ATE, VTE, HF, MI
Lenvatinib	2015	42	13	cardiomyopathy, HF, ATE, LQTS
Cabozantinib	2016	37–81	15–28	MI, ATE, VTE
Tivozanib	2021	44–45	12–22	HF, MI, ATE, VTE

*This table shows data about incidence of tHTN collected from FDA, Phase III clinical trials and meta-analysis or other high-grade evidences. The Grade 3/4 HTN data about Temsirolimus has not been found. HTN, hypertension; VTE, venous thromboembolism; HF, heart failure; ATE, arterial thromboembolism; MI, myocardial infarction; LQTS, long Q-T syndrome; TdP, Torsade de Pointes*.

### Nephrectomy-Related HTN

As for PN, a cross-sectional survey showed that PN was independently associated with NR-HT (*OR* = 2.93, *p* = 0.022) (8). There are several hypotheses for NR-HT after PN. The compressed renal parenchyma due to renal hematoma, bolsters, or sclerotic tissue could cause insufficient renal perfusion and renin-angiotensin system activation, which refers to the “page kidney” hypothesis (71). In addition, vascular clamping in PN process could cause vasculitis and intimal hyperplasia, which would aggravate renal artery stenosis, resulting in the decline of glomerular capillary pressure and activated rennin-angiotensin system, leading to NR-HT (72). However, some studies drew opposite conclusions (67, 73). A retrospective study involving 264 patients with PN showed that BP had no significant change after surgery (67). A plausible explanation is that PN may treat paraneoplastic HTN, which can mask NR-HT, thus resulting in a statistically insignificant difference. Another study showed the BP decreased 1.9 mmHg (*p* = 0.01) in 5 years after PN and the decrease of BP is thought to be associated with more BP measurements during follow-up and increased antihypertensive medications (73). Considering the conflicting results, well-designed prospective researches are warranted for NR-HT.

As for RN, it is still uncertain for its facilitating HTN role owing to insufficient evidence. After more than 10 years of follow-up, a small cohort study showed that 40% of patients with RN developed NR-HT and the mechanisms of RN leading to NR-HT are most likely due to functional renal parenchyma deficits and secondary end-stage renal disease (68). However, another cross-sectional cohort study showed that there was no significant difference in BP among RCC patients who underwent RN (69). Besides, the circadian rhythm of BP may also be affected after bilateral RN (74).

### Targeted Treatment-Related HTN

Targeted therapies for mRCC have prolonged the overall survival (OS) and progression-free survival (PFS) significantly and now have been listed as the standard treatment for mRCC (3). However, the number of patients with mRCC complicated with targeted therapy-related HTN (tHTN) as the on-target effect has dramatically increased (75).

These targeted drugs for mRCC mainly include VSP inhibitors and phosphatidylinositol-3-kinase–protein kinase B/mammalian target of rapamycin (mTOR) inhibitors. Bevacizumab is a monoclonal antibody to VEGF, often accompanied by the use of IFN-α (3). Multitargeted tyrosine kinase inhibitors (TKIs), which can bind to VEGF receptors and suppress the VEGF pathway, include sunitinib, sorafenib, pazopanib, axitinib, tivozanib, and cabozantinib (76). The mTOR inhibitor includes everolimus and temsirolimus (3). According to a report of real-world treatment patterns, the most common first-line used of targeted drugs in 2015 in the United States are sunitinib and pazopanib accounting for about 70% (77).

Strong evidence showed that targeted therapy, especially VSP inhibitors, could induce HTN. We collect data about tHTN from FDA (70), Phase III clinical trials, meta-analysis, or other high-grade evidence RCC (78–84) (Table 1). The Common Terminology Criteria for Adverse Events classified the tHTN into 5 grades. A meta-analysis of randomized controlled trials in 2015 showed that patients with TKIs have a significantly higher grade 3 or 4 HTN incidence compared with IFN-α or placebo (*RR* = 6.00, 95% *CI*: 3.36–10.69) (9). A large retrospective real-world study from 2006 to 2015 showed the total tHTN incidence rate was 69.1 per 100 patient-years and VSP inhibitors were higher than mTOR inhibitors (71.7 vs. 47.8 per patient-years) (77). The newer generation of VSP inhibitors which are more powerful to inhibit the VEGF pathway, tended to have higher HTN incidence (85). In addition, higher doses and longer duration of VSP inhibitors will increase the incidence and degree of HTN, which showed a dose-dependent relationship (2, 85, 86). Germline polymorphisms (86), high SBP at baseline (87), aging and other cardiovascular risk factors (88) may also affect the onset of tHTN. The tHTN could occur within hours or days after receiving VSP inhibitors (9) and drop quickly after drug withdrawal (89). The average onset time of tHTN was 131 days for bevacizumab (78), 116.5 days for mTOR inhibitors, and 70.0 days for VSP inhibitors (77). The newly proved lenvatinib has a median onset time of 35 days, reported by the FDA (70). The use of antihypertensive drugs may affect the onset time of severe HTN (9).

The mechanisms for tHTN are still elusive. VSP inhibitors could cause depletion of nitric oxide and prostacyclin which are vasodilators as well as increased vasoconstrictive endothelin-1 (89). In addition, increased reactive oxygen species, functional decreased microvascular density, increased vascular stiffness, and salt sensitivity are other possible reasons (90).

The tHTN could be seen as a biomarker for the on-target effect of VSP inhibitors and indicated a better prognosis (75). A multicenter retrospective study in 2020 demonstrated that patients with tHTN had higher PFS (12 months, 95% *CI* = 9–21 months) than those without tHTN (9 months, 95% *CI*: 7–12 months) (86). Similar results were shown among other TKIs and Bevacizumab (2, 75).

However, HTN that is not induced by targeted therapy could increase the risk of RCC mortality (*OR* = 1.75, 95% *CI*: 1.61–1.90) demonstrated by a review such as 6,964 patients of RCC in 2002 (91). Severe HTN in patients of RCC could cause HF, leukoencephalopathy, suspend, or cessation of targeted drugs (75, 90), which will do harm to the prognosis and well-control of BP during targeted therapy could improve prognosis (92).

### Selection of Antihypertensive Drugs for tHTN

There is no conclusion about the best antihypertensive drugs for tHTN (81). The current opinion is the selection of antihypertensive drugs should be individualized, but there are indeed some preferences (93).

Angiotensin-converting enzyme inhibitors/ angiotensin receptor blockades are potential better antihypertensive drugs for VSP inhibitors users. Several retrospective studies showed that patients of mRCC treated with sunitinib or other VSP inhibitors had better OS and PFS if received ACEI/ARBs (92, 94). ACEI/ARBs may be more recommended in patients with mRCC undergoing nephrectomy, considering its renal protective function (95). ACEI/ARBs could also treat proteinuria and left ventricular systolic dysfunction induced by targeted treatment (81, 90). ACEI/ARBs may prevent sarcopenia in patients with RCC and then reduce overexposure and toxicity of TKIs which could decrease the treatment interruption rate (95). However, a case report claimed that ACEI may decrease the effect of bevacizumab in ovarian cancer (96) and another case reported that combinatorial therapy of ACEI and everolimus may increase the risk of angioedema (97). A pooled-analysis reported that baseline use of ACEI/ARB is not significantly associated with OS or PFS (81). Thus, even with much supporting evidence, the priority of ACEI/ARB in mRCC needs further studies.

Dihydropyridine CCB can control tHTN as well as other antihypertensive drugs, considering the function of inhibiting arterial wall contractility (98). In addition, CCB was thought to inhibit chemoresistance of RCC and thus enhance drug efficacy (88). Besides, animal studies showed that CCB could increase the density of micro-vessels (89). But non-dihydropyridine CCB should not be used in patients receiving VSP inhibitors because they would competitively inhibit the activity of P450 3A4, thus increasing the circulating VSP inhibitors concentrations (2).

A retrospective study showed that the patients with mRCC treated with sunitinib or pazopanib with β-blockers have better PFS and OS than other antihypertensive drugs (99). Animal studies have shown that β-blockers could inhibit the proliferation of cancer, but the anti-cancer role of β-blockers in human is still in controversy (100).

The use of diuretics should consider the probability of dehydration and electrolyte disorders, since patients treated with VSP inhibitors like sunitinib have the higher risk of diarrhea and electrolyte imbalances (95). Fluid retention due to sodium excretion depletion may explain tHTN occurred weeks later and diuretics are a potential preference in this condition (101).

## Discussion

### What Are the Risk Factors for Comorbidity of HTN and RCC?

The relationship between HTN and RCC is complex (Figure 1). HTN and RCC share several common modifiable risk factors, such as obesity, smoking, and low physical activity. These risk factors may induce RCC and HTN through several common mechanisms, for example, chronic inflammation, oxidative stress like lipid peroxidation, interleukin-6, insulin, IGF-1, leptin, and VEGF pathway (48). There are also some potential common risk factors, like unhealthy diet, alcohol, but need further study to confirm their roles.

Hypertension is a direct risk factor for RCC with a dose-dependent relationship. HTN may also play a synergistic role with other risk factors like obesity to facilitate RCC. Meanwhile, the risk of RCC caused by antihypertensive drugs has not been excluded and diuretics are with great suspicion to cause RCC. Notably, ACEI/ARBs are potential anti-cancer drugs considering their mechanisms of function.

Renal cell carcinoma can directly cause HTN by the formation of arteriovenous fistula, tumor compression-induced renin secretion, ectopic hormone syndromes, paraneoplastic vasculitis, and brain metastasis. Treatment of RCC can also induce HTN. Nephrectomy may affect BP. The use of targeted therapy is strongly associated with HTN. This kind of increased BP is short-term, reversible, and dose-dependent and indicates the effect of targeted therapy. As to medicine for tHTN, there is no strong evidence proving a preference for a certain kind of antihypertensive drugs.

There are some guiltless factors responsible for the increasing comorbidity. For example, population growth and aging, advances in cancer treatment and prolonged survival, widespread use of advanced imaging techniques, improved public awareness for annual medical examination (44, 48, 102).

### How to Decrease the Comorbidity of HTN and RCC?

**Figure 2:**
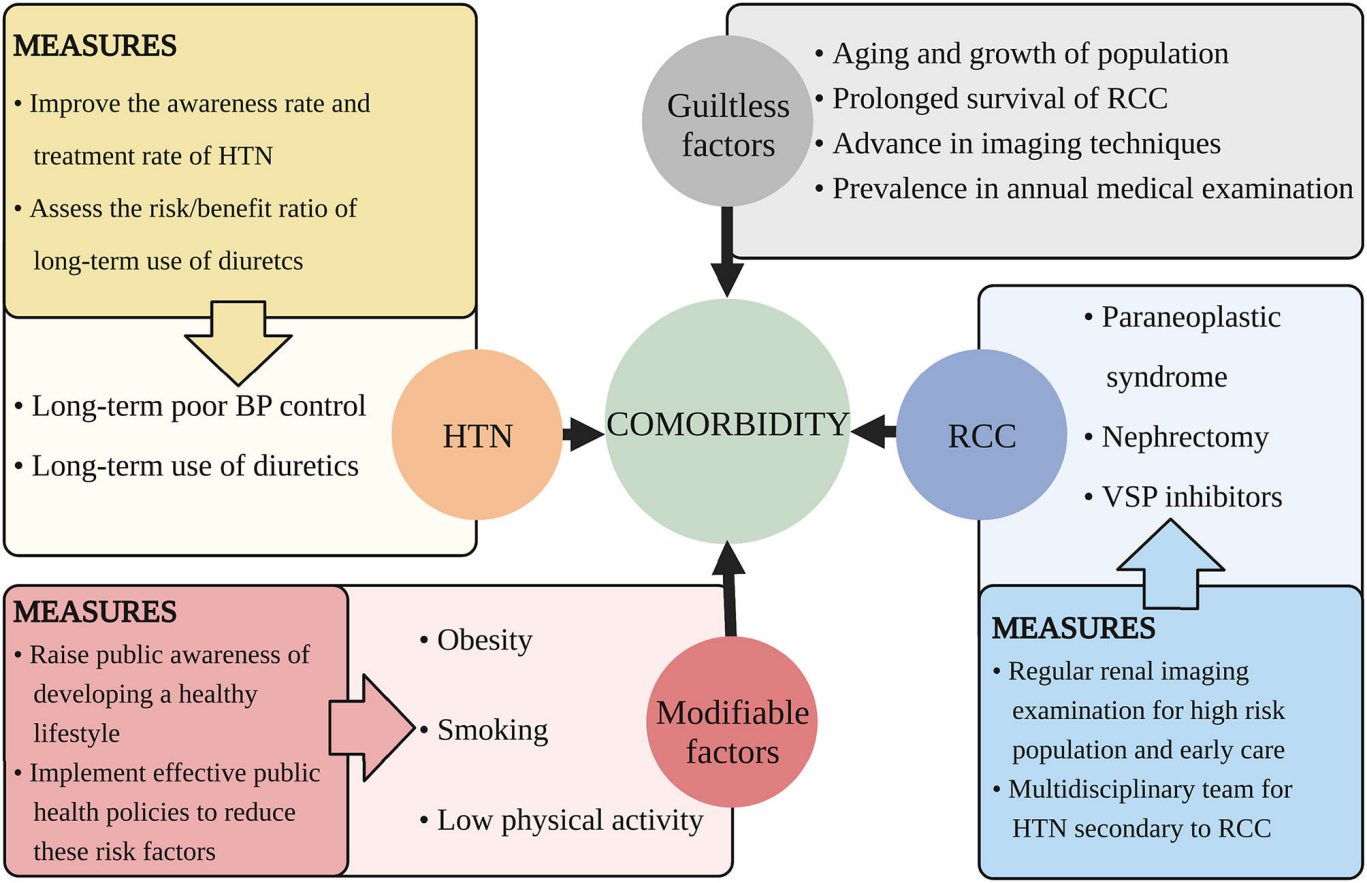
Suggested measures for decreasing the comorbidity of HTN and RCC. This figure illustrates aspects that cause the increasing comorbidity of HTN and RCC and proposes related measures. HTN, hypertension; RCC, renal cell carcinoma; BP, blood pressure; VSP, vascular endothelial growth factor signaling pathway.

There are some factors that we can handle to decrease the comorbidity and the suggestions are discussed below (Figure 2).

Obesity, smoking, and lack of physical activity all show a dose-dependent relationship with RCC and HTN. Obesity was supposed to be associated with 78% patients of HTN, and increased 60% risk of RCC (90). The risk of RCC doubled in people smoking more than 20 cigarettes a day while cessation of smoking more than 30 years reduced the 50% risk of RCC (32). Adequate physical activity can reduce 12% risk of RCC (37). It is known that prevention is more favorable than treatment, both for patients and for society. Thus, public awareness of developing a healthy lifestyle should be raised and effective public health policies should be implemented to reduce the modifiable risk factors.

Poor control of essential HTN facilitates the rising prevalence of RCC. Globally, it is estimated that only half patients of HTN are diagnosed and one-fifth patients of HTN have well-controlled BP (1). Thus, improvement of the awareness and treatment rate of HTN is quite urgent.

Diuretics exert a potential carcinogenic effect, thus it should be prescribed after comprehensive thought of the risk/benefit ratio (103). For those with severe HF, refractory HTN, or edema, the benefit is higher than the risk. Considering the carcinogenic risk of diuretics is low and needs long-term accumulation to be significant, younger women who need decades of use of diuretics are at high risk than the elderly and if not necessary, better change to other antihypertensive drugs.

For those with obesity, smoking exposure, low activity, and unhealthy diets, the risk of RCC or HTN is high. For those with long-term HTN and diuretics history, RCC will occur in higher possibility. People with malignant HTN also have a higher incidence of RCC (7). These groups with a high risk of RCC are recommended for regular renal imaging examination. And for those already diagnosed with RCC and HTN, cardio-oncologic teams are needed to give better clinical care.

### How to Give Better Antihypertensive Treatment for the Patients With RCC With HTN?

Regular and accurate BP measurement is fundamental. If the patients with RCC are treated with nephrectomy and targeted therapy, clinicians need to predict the possible changes of blood pressure and monitor BP regularly. As to tHTN, guideline recommend well-controlled BP before targeted treatment and weekly monitor during the first treatment cycle and monitor every 2–3 weeks in the remaining treatment cycle (76). For those with a history of HTN or coronary heart disease, the risk of cardiovascular event is significantly higher when receiving VSP inhibitors and should be monitored with caution (104). Hypotension may also occur as a manifestation of hypersensitivity/infusion reactions when receiving targeted therapy (70). Thus, BP should be monitored throughout the infusion process and necessary supportive care should be prepared. Notably, white-coat HTN, masked HTN may conceal the exact BP, so out-of-office measurements are also necessary (105).

Well-control of BP can improve the prognosis of RCC by preventing severe cardiovascular diseases or discontinuation of targeted drugs (104). The HTN caused by VSP inhibitors is usually mild and reversible (106). According to ACC/AHA guidelines, for people taking VSP inhibitors, the recommended BP is below 140/90 and below 130/80 if with cardiovascular risk factors (105). However, there is limited evidence to support this antihypertensive target. The patients with RCC with VSP inhibitors developed stage I HTN or DBP increased >20 mmHg should use antihypertensive drugs (107). The pros and cons of each kind of antihypertensive drugs have been discussed above. If the HTN could not be controlled well with a single agent, consider combined therapy methods. If the HTN is uncontrolled with end organ damage, the cessation of VSP inhibitors is recommended (108). Paraneoplastic HTN is usually reversible after renal tumor removal and there is a lack of evidence for antihypertensive therapies for NR-HT. Besides, better pain control and psychotherapy are necessary in the control of BP in the patients with RCC (2).

However, in view of the lack of high-level evidence for the management of HTN in the patients with RCC and different comorbidity conditions of patients, the strategy of blood pressure control is often best guided by a team of oncologist, cardiologist, and clinical pharmacist (108). It is necessary to improve the understanding of “cardio-oncology” among health professionals. The term “cardio-oncology” highlights the complex relationship between cardiovascular diseases and cancer, and encourages the corporation of cardiovascular specialists and oncologists to give better clinical care for cancer survivors.

## Conclusions

In modern society, owing to the change of lifestyle and use of VSPs, the number of patients with HTN and RCC simultaneously is increasing, which turns out to be a heavy disease burden. This review thoroughly investigated the relationship between RCC and HTN from basic, epidemiological, and clinical aspects, aiming to deepen the understanding of the comorbidity and benefit of these patients. However, many problems remain to be resolved. Apart from obesity, smoking, and low physical activity, there are still other possible common modifiable risk factors without robust evidence. Besides, the exact roles of antihypertensive drugs on tumor formation are uncertain and high quality evidence regarding the management of HTN secondary to RCC is far from enough to generate guidance for clinicians. Thus, we appealed to the corporation of basic scientists, public health officers, oncologists, cardiologists, and other health experts to solve these cardio-oncologic problems.

## Author Contributions

ZB and JY: conception and thoroughly searching related papers. ZB, YX, MH, DL, HW, HL, and JY: drafting of the manuscript or revising it critically for important intellectual content. YX and ZB: drawing illustrations. JY: final approval of the manuscript submitted. All authors contributed to the article and approved the submitted version.

## Conflict of Interest

The authors declare that the research was conducted in the absence of any commercial or financial relationships that could be construed as a potential conflict of interest.

## Publisher's Note

All claims expressed in this article are solely those of the authors and do not necessarily represent those of their affiliated organizations, or those of the publisher, the editors and the reviewers. Any product that may be evaluated in this article, or claim that may be made by its manufacturer, is not guaranteed or endorsed by the publisher.

## Abbreviations

ACEI: angiotensin-converting enzyme inhibitor
a-MSH: a-melanocyte-stimulating hormone
ARB: angiotensin receptor blockade
BMI: body mass index
BP: blood pressure
CCB: calcium channel blocker
DBP: diastolic blood pressure
HR: hazard ratio
HTN: hypertension
IGF-1: insulin-like growth factor-1
mRCC: metastatic RCC
mTOR: mammalian target of rapamycin
OR: odds ratio
OS: overall survival
PFS: progression-free survival
PN: partial nephrectomy
RCC: renal cell carcinoma
RR: relative risk
RN: radical nephrectomy
SBP: systolic blood pressure
tHTN: targeted therapy-related HTN
TKI: tyrosine kinase inhibitor
VEGF: vascular endothelial growth factor
VSP: vascular endothelial growth factor signaling pathway.
