# Supplementary material for: Risk Factors for the Comorbidity of Hypertension and Renal Cell Carcinoma in the Cardio-Oncologic Era and Treatment for Tumor-Induced Hypertension
Source: Front Cardiovasc Med. 2022 Feb 17;9:810262. doi: 10.3389/fcvm.2022.810262 (PMC8892205; doi:10.3389/fcvm.2022.810262)
Supplement: Supplementary file 1 [file Data_Sheet_1.docx]

# Search strategy

PubMed (1946-2021) and Cochrane Library (1996-2021) were employed as the source of initial searches. Hand searching was also used to find relevant studies in PubMed and other websites (e.g., FDA, SEER). Besides, valuable publications recommended by experts were included as well.

## Database: PubMed

**Query:**

(("Hypertension"[Mesh] OR "Hypertension, Renal"[Mesh] OR "Blood Pressure"[Mesh] OR "Arterial Pressure"[Mesh] OR hypertensive OR hypertension)

OR (antihypertensive OR "Antihypertensive Agents"[Mesh] OR "Antihypertensive Agents" [Pharmacological Action] OR "Vasodilator Agents"[Mesh] OR "Vasodilator Agents" [Pharmacological Action] OR "Adrenergic alpha-Antagonists"[Mesh] OR "Adrenergic alpha-Antagonists" [Pharmacological Action] OR "Adrenergic beta-Antagonists"[Mesh] OR "Adrenergic beta-Antagonists" [Pharmacological Action] OR "Sodium Chloride Symporter Inhibitors"[Mesh] OR "Sodium Chloride Symporter Inhibitors" [Pharmacological Action] OR "Angiotensin-Converting Enzyme Inhibitors"[Mesh] OR "Angiotensin-Converting Enzyme Inhibitors" [Pharmacological Action] OR "Renin-Angiotensin System"[Mesh] OR "Vasopeptidase Inhibitors"[Mesh] OR "Angiotensin II Type 1 Receptor Blockers "[Mesh] OR "Angiotensin II Type 1 Receptor Blockers" [Pharmacological Action] OR "Angiotensin Receptor Antagonists"[Mesh] OR "Angiotensin Receptor Antagonists" [Pharmacological Action] OR "Angiotensin II Type 2 Receptor Blockers"[Mesh] OR "Calcium Channel Blockers"[Mesh] OR "Calcium Channel Blockers" [Pharmacological Action] OR Renin-Angiotensin System OR "angiotensin receptor blocker" OR "angiotensin receptor antagonist" OR "angiotensin receptor blockade" OR "angiotensin-receptor blocker" OR "angiotensin-receptor antagonist" OR "angiotensin-receptor blockade" OR "renin-angiotensin system inhibitor" OR "ARB" OR "ARBs" OR angiotensin-converting enzyme inhibitor OR angiotensin-converting enzyme inhibitor* OR ACE inhibitor* OR ACEI* OR captopril OR ramipril OR cilazapril OR enalapril OR fosinopril OR perindopril OR imidapril OR lisinopril OR moexipril OR quinapril OR trandolapril OR diuretics OR diuretic*))

AND ("Kidney Neoplasms"[Mesh] OR "Carcinoma, Renal Cell"[Mesh] OR kidney cancer OR renal cell cancer kidney carcinoma OR renal cell carcinoma)

**Results:** 7,192

**Search date:** 2020-08-22

## Database: Cochrane Library

Search Name: HTN & RCC

Date Run: 22/08/2021 18:56:16

ID Search Hits

#1 MeSH descriptor: [Antihypertensive Agents] explode all trees 8247

#2 MeSH descriptor: [Hypertension] explode all trees 19408

#3 MeSH descriptor: [Carcinoma, Renal Cell] explode all trees 1008

#4 (#1 or #2) and #3 13

## Other Sources:

By using hand searching strategy in PubMed and other websites (e.g., FDA, SEER). Experts recommended publications. Results: Found 74 studies.

# Screening and eligibility:

6441 studies were excluded for not meeting the inclusion criteria. 715 studies were excluded for not meeting the exclusion criteria. The review process is conducted independently by 3 authors. Discrepancies were solved by consensus.

**Inclusion Criteria ：**

1) epidemiology about RCC or HTN;

2) risk factors causing RCC or HTN;

3) mechanisms for formation of RCC or HTN;

4) treatment of HTN in RCC patients.

**Exclusion Criteria：**

1) not in English;

2) duplicate;

3) clinical studies that have similar results but with lower evidence level or out of date;

4) could not find full text.

**Identification of studies via databases**

Records removed *before screening*:

Duplicate records removed

(n = 15)

Records identified from:

PubMed (n = 7192)

Cochrane Lib (n = 13)

Other Sources (n = 74)

**Identification**

Records screened

(n = 7264)

Records excluded

(n = 6441)

Reports sought for retrieval

(n = 823)

Reports not retrieved

(n = 0)

**Screening**

Reports assessed for eligibility

(n = 823)

Reports excluded for meeting the exclusion criteria (n = 715)

Studies included in review

(n = 108)

**Included**

*From:*  Page MJ, McKenzie JE, Bossuyt PM, Boutron I, Hoffmann TC, Mulrow CD, et al. The PRISMA 2020 statement: an updated guideline for reporting systematic reviews. BMJ 2021;372:n71. doi: 10.1136/bmj.n71
